# Gastrointestinal Stromal Tumor (GIST): A Remarkable Case Report and Literature Review

**DOI:** 10.7759/cureus.35931

**Published:** 2023-03-09

**Authors:** Bassey Enodien, Dana Hendie, Tobias Müller, Stephanie Taha-Mehlitz, Daniel M Frey, Anas Taha

**Affiliations:** 1 Department of Surgery, GZO Hospital, Wetzikon, CHE; 2 Department of Surgery, College of Medicine, Sulaiman Al Rajhi University, Albukairyah, SAU; 3 Clarunis, Department of Visceral Surgery, University Center for Gastrointestinal and Liver Diseases, St. Clara Hospital and University Hospital Basel, Basel, CHE

**Keywords:** review, colorectal gist, gastrointestinal stromal tumor (gist), perforation, gist

## Abstract

Gastrointestinal stromal tumor (GIST) makes up less than 1% of all gastrointestinal tumors, but it is the most common mesenchymal tumor of the digestive system. It is commonly found in the stomach and the small intestine and rarely seen in the colon and the esophagus. Additionally, sigmoid GIST is quite rare since colorectal GIST often occurs in the rectum. A total of 21 patients (including the study case) were looked at for this study, of which 14 (66.6%) were males and seven (33.3%) were females. We focused on GIST and conducted an online search and systematic analysis of all case presentations.

## Introduction

Gastrointestinal stromal tumor (GIST) is one of the most prevalent mesenchymal tumors of the digestive tract despite constituting less than 1% of all gastrointestinal tumors. Historically, it was thought to originate from smooth muscle and misclassified as leiomyoma, leiomyoblastoma, and leiomyosarcoma [1]. However, due to modern advancements in molecular technique, it was discovered that GIST originates from the interstitial cells of Cajal or a precursor cell [2]. It was also uncovered that it was caused most importantly by an oncogenic mutation in the *KIT* gene, which is responsible for the regulation of tyrosine kinase [3]. Most GISTs usually occur in the stomach followed by the small intestine and rarely in the colon and esophagus. Furthermore, colorectal GIST is usually in the rectum, so sigmoid GIST is rather uncommon [4].

## Case presentation

**Figure 1:**
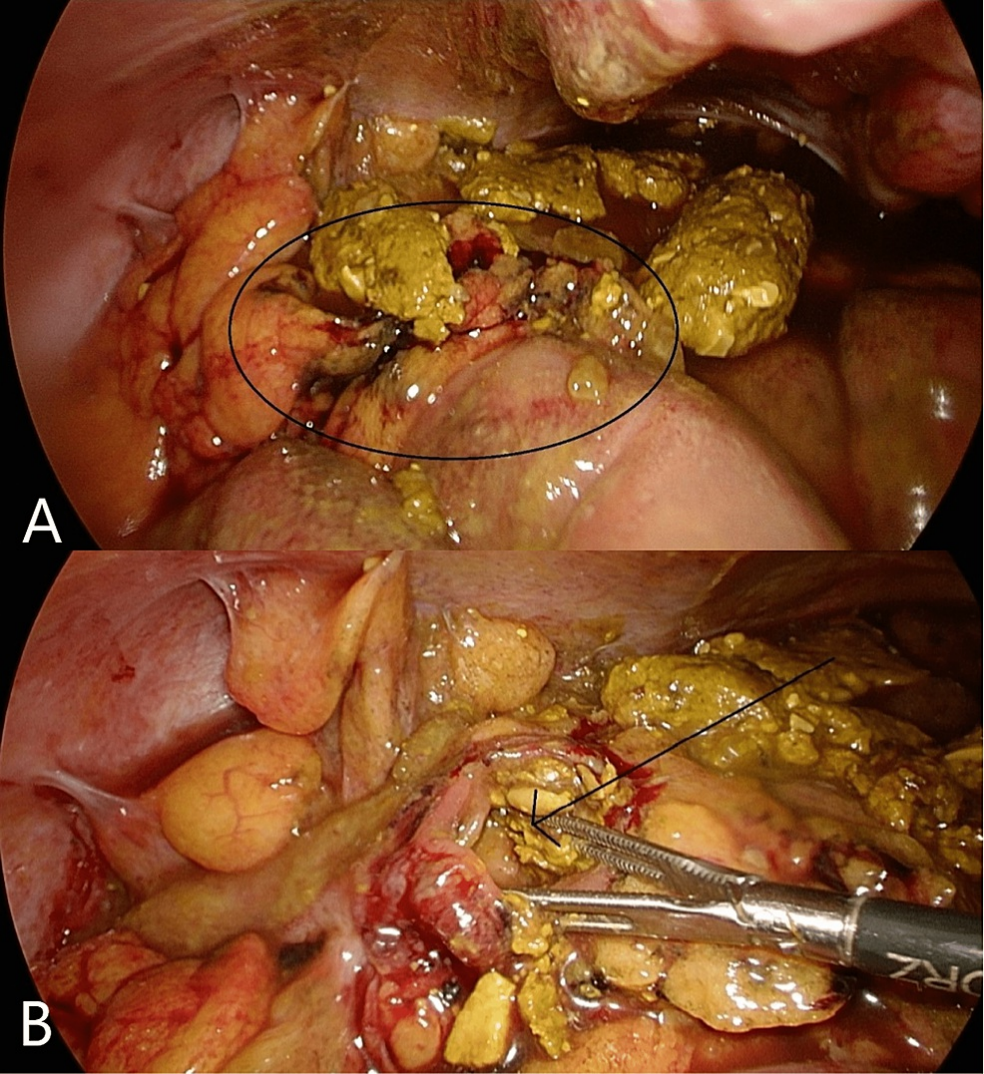
A laparoscopic view that reveals widespread peritonitis and widespread feces. A) A 1.5 cm sigmoid colon perforation. B) Little tumor (arrow) adjacent to the perforation

A 74-year-old male with a background of chronic obstructive pulmonary disease presented to the emergency department with a sudden onset of severe abdominal pain located in the lower abdomen. Moreover, despite normal laboratory results, the patient’s pain did not abate. An abdominal computed tomography (CT) scan was performed, and it illustrated a mid to lower intestinal perforation, suspected to be from either the sigmoid or the small bowl. Moreover, a diagnostic laparoscopy was done. The examination revealed diffuse peritonitis, fecal soiling of the entire abdomen, a 1.5 cm sigmoid colon perforation, and what appeared to be a small tumor close to the site, as can be seen in Figure 1.

Foremost, the removal of fecal material and the irrigation of the entire abdomen were initiated. Thereafter, laparoscopic partial sigmoid resection, i.e., Hartmann procedure, was done, and the tumor was then sent to for histological examination. The tumor is shown to express cluster of differentiation (CD) 117 and discovered on gastrointestinal stromal tumor protein 1 (DOG-1) confirming the diagnosis of a GIST, measuring at 0.7 cm with a low mitotic rate. There was no evidence of lymphatic or perineural sheath infiltration. The tumor’s tumor-node-metastasis (TNM) classification was pT1 L0 V0 Pn0 R0. Microscopically, the tumor had a spindle-like morphology with no necrosis nor high-grade atypia. It is also pertinent to note that the patient has experienced a myriad of complications following surgery including sigmoid stump insufficiency, multiple abscesses, urinary retention, postoperative ileus, intermittent tachycardic atrial fibrillation, and recurrent pleural effusion. The patient’s complications were treated over a period of several weeks, and he was later discharged. It was determined that the patient required follow-up examinations every 6-12 months. However, the patient refused chemotherapy, so annual CT scans are done regularly.

Methods

**Figure 2:**
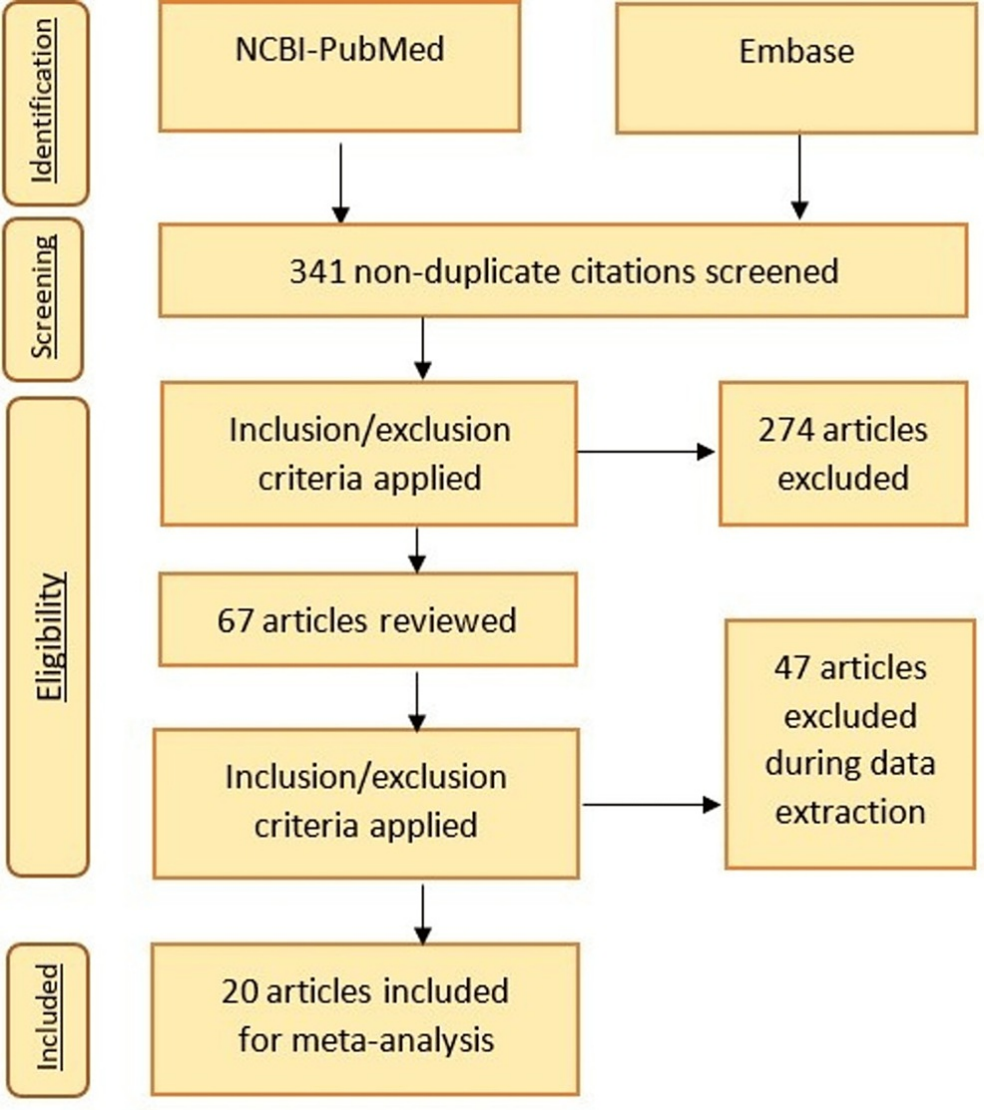
PRISMA diagram depicting the flow of information across the various phases of a systematic review. NCBI, National Center for Biotechnology Information; PRISMA, Preferred Reporting Items for Systematic Reviews and Meta-Analyses

We searched the internet for data on GIST and systematically analyzed all case reports from September 16, 2022, to October 6, 2022. Using the search phrases “Gastrointestinal stromal tumour case report,” all studies were located on National Center for Biotechnology Information (NCBI)-PubMed (www.pubmed.gov) and Embase (www.embase.com). The inclusion criteria were determined to be patients suffering from GIST, patients of both sexes, patients of various ages, patients of various body weights, and patients of various particular medically significant habits. Furthermore, the exclusion criteria were patients with comorbidities that could raise their risk for adverse events or affect the study’s findings, patients with terminal illnesses, patients with more than one tumor, or patients with a tumor other than the GIST, as well as patients who are likely to be lost to follow-up, missed data collection interviews, or gave inaccurate information. We looked at all studies applicable in English (or those having relevant English-language abstracts). The search results were as follows: There were 341 articles (not duplicates), of which 274 papers were disregarded because they did not primarily address GIST in their research title or abstract. Additionally, 47 publications were disregarded throughout the data extraction process after applying the inclusion and exclusion criteria. The other 20 papers were all then added to this study, and they were all carefully evaluated and assessed, as seen in Figure 2.

Result

According to our research of the literature, a total of 21 patients (including the study case) were investigated, of which 14 (66.6%) were males and seven (33.3%) were females. Their ages varied from 30 to 85 years, with a mean age of 64.05. The vast majority of patients (61.9%) were above the age of 60. The majority of cases demonstrate that the tumor had spindle cells with a high rate of mitosis. Additionally, immunocytochemistry shows that the majority of patients have a strong positivity to certain antibodies such as CD117, DOG-1, CD34, and vimentin being expressed, as seen in Table 1.

**Table 1:** The clinical features and an overview of the case reports presenting gastrointestinal stromal tumor (GIST). SDHB, succinate dehydrogenase B; SMA, smooth muscle antibody; NSE, neuron-specific enolase; MCT, mast cell tryptase; CD, cluster of differentiation; ALK, anaplastic lymphoma kinase; BCL-2, B-cell lymphoma 2; CKAE1, anticytokeratin monoclonal antibody 1; WT1, Wilms’ tumor-1; AE3, monoclonal antibody 3; CKP, cytokeratin-positive gastrointestinal stromal tumor; HMB45, human melanoma black-45

Article	Age/years	Gender	Complaint	Site	Antibodies expressed	
Present case	74 years	Male	Presented to the emergency department (ED) with severe abdominal pain	Sigmoid colon	CD117 (+) and DOG-1 (+)	
Yuan et al., 2021 [5]	71 years	Male	Reported to the emergency room with a 38-hour history of intermittent hematochezia and diffuse abdominal pain	Small intestine (third jejunal segment)	CD117 (+), DOG-1 (+), SDHB (+), desmin (+), actin (+), CD34 (-), and S-100 (-)	
Yang et al., 2022 [6]	59 years	Male	The patient’s symptoms are consistent with incomplete obstruction: the patient felt unclean and noticed a shift in bowel habits with more frequent stools with a decrease in single stool volume	Rectum	CD117 (+), DOG-1 (+), CD34 (+), and Ki67 (+)	
Tezcan and Koç, 2011 [7]	83 years	Male	Complaint of constipation and then one year later complaint of pain in the right hip	Rectum	CD117 (-), CD34 (-), S-100 (-), SMA (-), and desmin (-)	
Sugimoto et al., 2013 [8]	52 years	Male	Complaint of anorexia and physical exhaustion	Upper stomach	CD117 (+), CD34 (+), S-100 (-), SMA (-), and desmin (-)	
Manxhuka-Kerliu et al., 2014 [9]	30 years	Female	Presented with nausea, vomiting, and abdominal pain	Small intestine	CD117 (+), CD34 (+), vimentin (+), actin (+ focally), desmin (-), and S-100 (-)	
Cheng et al., 2019 [10]	78 years	Female	Referred to the hospital with 10 days of ongoing vaginal bleeding and difficulty urinating	Cervical or rectal mass with rectovaginal invasion		CD117 (+), DOG-1 (+), H-caldesmon (+), actin and SMA (-), p40 (-), and S-100 (-)
Liu et al., 2018 [11]	72 years	Male	Complaint of upper abdominal pain for one month with regurgitation and weight loss	Gastric fundus	CD117 (+), vimentin (+), CD34 (+), S-100 (+), SMA (+), WT1 (-), Ki67 (-), and BCL-2 (-)	
Wang et al., 2017 [12]	74 years	Female	Hospitalized as a result of worsening abdominal pain for two days with history or three months of abdominal pain and distention	Gastric fundus	CD117 (+), H-caldesmon (+), DOG-1 (+), SMA (weak +), CD34 (-), S-100 (-), desmin (-), and NSE (-)	
Lech et al., 2015 [13]	52 years old	Male	Reported losing about 5 kg in the last two weeks along with frequent heartburn and overall malaise	Posterior gastric wall	CKAE1 (+), vimentin (+), CD117 (+/-), CD34 (-), CD30 (-), AE3 (-), S-100 (-), H-caldesmon (-), actin (-), MCT (-), and fat (-)	
Xu et al., 2015 [14]	53 years old	Male	History of intermittent abdominal pain and bloating without any constitutional symptoms	Mesentery of the small intestine	CD117 (c-KIT) (+), vimentin (+), CD34 (partial +), CD68 (partial +), DOG-1 (-), lysozyme (-), HMB45 (-), S-100 (-), and cytokeratin (-)	
Yuval et al., 2014 [15]	64 years old	Female	Presented with melena and syncope to the emergency department (ED) (hypovolemia)	Jejunum	CD117 (+)	
Misawa et al., 2014 [16]	70 years old	Male	Presented with a fever and abdominal pain	Jejunum	CD117 (+) and CD34 (+)	
Ma et al., 2017 [17]	56 years old	Male	Presented with a 24-hour melena history	Jejunum	CD117 (+), CD34 (+), and DOG-1 (+)	
Skipworth et al., 2014 [18]	51 years old	Female	A one-day history of acute, sudden-onset epigastric pain	Gastric antrum	DOG-1 (+) and CD117 (-)	
Niazi et al., 2014 [19]	55 years old	Female	Presented with a vague abdominal pain; various investigations were negative. She experienced acute abdominal pain a year later	Rectosigmoidal mass	CD117 (+), CD34 (+), SMA (-), S-100 (-), ALK (-), beta-catenin (-), AE1/3 (-), and desmin (equivocal)	
Iusco et al., 2010 [20]	76 years old	Male	Presented with intestinal subocclusion symptoms	Ileum	CD117 (+), CD34 (weak+), actin (-), and S-100 (-)	
Đokić et al., 2019 [21]	62 years old	Male	Presented to the emergency room with severe epigastric pain that persisted for days and black stool lasting for a week with vomiting, inappetence, and weight loss	Lesser curvature of the gastric body	CD117 (+) and DOG-1 (+)	
Sahin et al., 2014 [22]	62 years old	Male	Referred to the hospital two years prior with abdominal pain, nausea, and vomiting	Gastric antrum	CD117 (+) and CD34 (+)	
Zhang et al., 2022 [23]	85 years old	Male	Presented to the hospital after experiencing frequent dark stools for 1.5 years	Gastric antrum	CD117 (weak +), CD34 (+), DOG-1 (+), SDHB (+), and CKP (-)	
Zhou et al., 2012 [24]	66 years old	Female	Presented with an aggravated abdominal ache that had been persistent for a year and was mostly accompanied by distension, eructation, epigastric fullness, decrease of food intake, and early satiety	Lesser curvature of the stomach	CD117 (+), CD34 (+), DOG-1 (+), Ki67 (+), and S-100 (-)	

**Figure 3:**
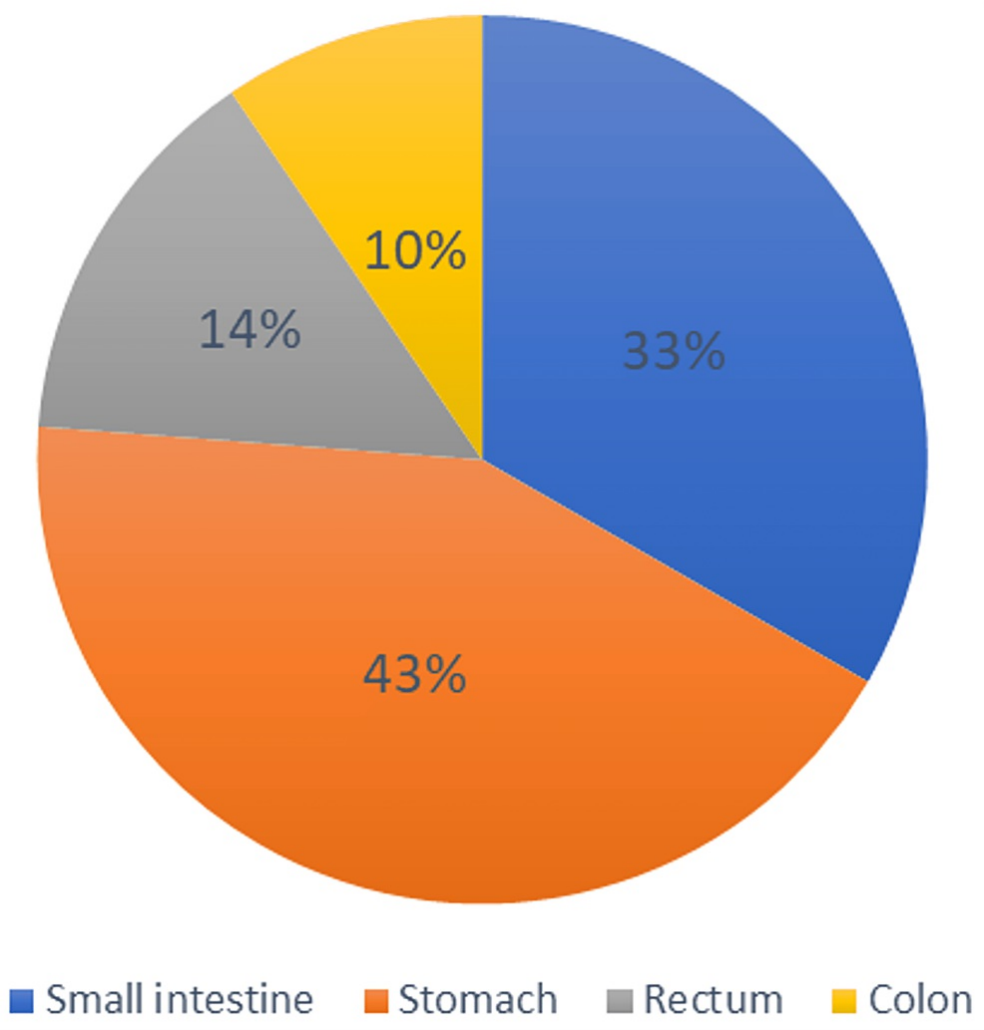
The percentage of the research population’s tumor sites.

These individuals’ medical histories were examined, and it was discovered that the majority of them complained of vague symptoms that may have misled the diagnosis. Complaints vary from constipation, exhaustion, nausea, and vomiting and can even progress to acute severe pain, shock, or other emergencies. The average tumor size in these cases is 8.39 cm. The data that was collected also revealed the tumor’s site, as seen in Figure 3.

## Discussion

Despite GIST being the most common gastrointestinal mesenchymal tumor, its precise and exact global incidence is still under consideration due to the absence of a complete definition and classification. However, it is estimated that 10-20 million people are afflicted by it [1]. With patients as young as 10 and as old as 100, the age distribution can vary greatly; the average age was found to be in the mid-60s. The gender distribution is equal. It is generally believed that the stomach (55.6%) and small intestine (31.8%) are the most prevalent sites for GIST, with the colon (6.0%) and esophagus (0.7%) being uncommon sites [25]. In regard to colonic GIST specifically, it is believed that the most common location is the sigmoid colon followed by the transverse colon, the descending colon, the ascending colon, and then the cecum [26].

Symptoms that lead to the diagnosis of a GIST are often vague, such as abdominal pain and bloating [3]. More specifically, gastric and small intestinal tumors cause ambiguous symptoms; occasionally, it presents as upper gastrointestinal tract bleeding and pain [4]. On the other side, bleeding and blockage may be observed with colorectal GIST. Additional early signs include pelvic and rectal pain, constipation, blockage, or a tumor on physical examination of the rectal cavity. Insidious signs of colorectal GIST include anemia, weight loss, and urinary symptoms including diminished stream strength or hesitation [27]. GIST can also be completely asymptomatic and found only incidentally during scans or physical examination. A small fraction of GIST can also be malignant [4]. GIST stretches widely along the array of malignant protentional at all locations of occurrence. Tumors that exhibit low mitotic rate of less than five mitoses per 50 high-power fields (HPF) are often benign, but there is definitely a percentage among them that metastasize [28]. However, a combination of small tumor that is less than 5 cm with a low mitotic rate is relatively enough to designate a tumor as low risk [4]. Moreover, large tumors of more than 5 cm in size and more than five mitoses per 50 HPF are considered of higher risk. High-grade malignant GIST, which are more than 50 mitoses per 50 HPF, can metastasize commonly intra-abdominally or to the liver. However, it is critical to emphasize that no lesion can be absolutely regarded as benign regardless of size and mitotic rate.

In regard to anatomical location, there is no consensus if it is a reliable predictor of malignant behavior [28]. GIST often appears grossly as an exophytic growth, appearing as a mass. Moreover, it is well-defined with a pseudo capsule and a smooth, gray, and white tint. Rarely, necrosis, bleeding, and cystic degeneration may be present [29]. Microscopically, GIST can appear as either one of the following groups: spindle cell (70%), epithelioid (20%), or mixed type (10%) [28]. Moreover, it was observed that anorectal tumors mostly fall in the spindle cell group, while surprisingly, colonic GISTs usually fall in the epithelioid category [27]. GIST is usually diagnosed by a myriad of antibodies including CD34, CD117, and DOG-1 [2]. The golden standard treatment of GIST regardless of location is surgical resection; usually, en bloc resection is done as GIST attaches to the surrounding organs and is very fragile [29]. However, surgery does not negate the risk of recurrence, which can be as high as 50%. Various chemotherapies were investigated due to this high recurrence rate, which led eventually to the discovery of imatinib, due to its natural resistance for conventional chemotherapy. This drug can be utilized after surgery in high-risk patients, in cases of metastasis or an unresectable tumor, as well as neoadjuvant therapy [27]. Imatinib can be substituted with other medications such as sunitinib and regorafenib. With varying degrees of success, newer therapeutics are still being researched, including endoscopic ultrasound-guided alcohol injection for GIST and several immunotherapies such nivolumab and ipilimumab [30]. Patients must be monitored for the rest of their lives.

## Conclusions

GIST is the most frequent gastrointestinal mesenchymal tumor; however, due to the lack of a comprehensive definition and classification, its true global incidence is still being debated. According to the literature, in our analysis of case reports, we have found that most GISTs are located in the stomach and are spindle-shaped. Moreover, most of these tumors were over 5 cm in size and have a high mitotic rate. GIST frequently exhibits symptoms, but the majority of these symptoms are vague and might mislead the diagnosis. This can result in sudden severe pain, shock, and other symptoms such as constipation, tiredness, nausea, and vomiting. Resection is the gold standard treatment for GIST and is typically followed by chemotherapy. Also, patients typically need lifelong follow-up.

## References

[1] Gupta P, Tewari M, Shukla HS (2008). Gastrointestinal stromal tumor. Surg Oncol.

[2] Mantese G (2019). Gastrointestinal stromal tumor: epidemiology, diagnosis, and treatment. Curr Opin Gastroenterol.

[3] Eisenberg BL, Pipas JM (2012). Gastrointestinal stromal tumor--background, pathology, treatment. Hematol Oncol Clin North Am.

[4] Miettinen M, Lasota J (2001). Gastrointestinal stromal tumors--definition, clinical, histological, immunohistochemical, and molecular genetic features and differential diagnosis. Virchows Arch.

[5] Yuan Y, Ding L, Tan M, Han AJ, Zhang X (2021). A concealed inguinal presentation of a gastrointestinal stromal tumor (GIST): a case report and literature review. BMC Surg.

[6] Yang J, Liu Y, Sun XJ, Ai ZW, Liu S (2022). A rare rectal gastrointestinal stromal tumor with indolent biological behavior: a case study. Exp Ther Med.

[7] Tezcan Y, Koç M (2011). Gastrointestinal stromal tumor of the rectum with bone and liver metastasis: a case study. Med Oncol.

[8] Sugimoto M, Hikichi T, Shioya Y (2013). A case of gastrointestinal stromal tumor with pneumomediastinum. Fukushima J Med Sci.

[9] Manxhuka-Kerliu S, Sahatciu-Meka V, Kerliu I, Juniku-Shkololli A, Kerliu L, Kastrati M, Kotorri V (2014). Small intestinal gastrointestinal stromal tumor in a young adult woman: a case report and review of the literature. J Med Case Rep.

[10] Cheng M, Liu CH, Horng HC, Chen YJ, Lo PF, Lee WL, Wang PH (2019). Gastrointestinal stromal tumor presenting as a rectovaginal septal mass: a case report and review of literature. Medicine (Baltimore).

[11] Liu S, Liu H, Dong Y, Wang F, Wang H, Chen J (2018). Gastric carcinoma with a gastrointestinal stromal tumor - a case report and literature review. Med Sci (Paris).

[12] Wang L, Liu L, Liu Z, Tian Y, Lin Z (2017). Giant gastrointestinal stromal tumor with predominantly cystic changes: a case report and literature review. World J Surg Oncol.

[13] Lech G, Korcz W, Kowalczyk E, Guzel T, Radoch M, Krasnodębski IW (2015). Giant gastrointestinal stromal tumour of rare sarcomatoid epithelioid subtype: case study and literature review. World J Gastroenterol.

[14] Xu L, Wen G, Ding Y, Zhao L (2015). A lethal mesenteric gastrointestinal stromal tumor: a case report and review of the literature. Int J Clin Exp Pathol.

[15] Yuval JB, Almogy G, Doviner V, Bala M (2014). Diagnostic and therapeutic approach to obscure gastrointestinal bleeding in a patient with a jejunal gastrointestinal stromal tumor: a case report. BMC Res Notes.

[16] Misawa S, Takeda M, Sakamoto H, Kirii Y, Ota H, Takagi H (2014). Spontaneous rupture of a giant gastrointestinal stromal tumor of the jejunum: a case report and literature review. World J Surg Oncol.

[17] Ma C, Hao SL, Liu XC (2017). Supraclavicular lymph node metastases from malignant gastrointestinal stromal tumor of the jejunum: a case report with review of the literature. World J Gastroenterol.

[18] Skipworth JR, Fanshawe AE, West MJ, Al-Bahrani A (2014). Perforation as a rare presentation of gastric gastrointestinal stromal tumours: a case report and review of the literature. Ann R Coll Surg Engl.

[19] Niazi AK, Kaley K, Saif MW (2014). Gastrointestinal stromal tumor of colon: a case report and review of literature. Anticancer Res.

[20] Iusco D, Jannaci M, Grassi A, Bonomi S, Ismail I, Navarra G, Virzì S (2010). Giant ileal gastrointestinal stromal tumour presenting as an intestinal subocclusion and subsequent haemoperitoneum: a case report and a review of the literature. Updates Surg.

[21] Đokić M, Novak J, Petrič M, Ranković B, Štabuc M, Trotovšek B (2019). Case report and literature review: patient with gastroduodenal intussusception due to the gastrointestinal stromal tumor of the lesser curvature of the gastric body. BMC Surg.

[22] Sahin E, Yetişyiğit T, Oznur M, Elboğa U (2014). Gastric gastrointestinal stromal tumor with bone metastases - case report and review of the literature. Klin Onkol.

[23] Zhang W, Chen H, Zhu L, Kong Z, Wang T, Li W (2022). Gastroduodenal intussusception caused by gastric gastrointestinal stromal tumor in adults: a case report and literature review. J Int Med Res.

[24] Zhou L, Liu C, Bai JG (2012). A rare giant gastrointestinal stromal tumor of the stomach traversing the upper abdomen: a case report and literature review. World J Surg Oncol.

[25] Søreide K, Sandvik OM, Søreide JA, Giljaca V, Jureckova A, Bulusu VR (2016). Global epidemiology of gastrointestinal stromal tumours (GIST): a systematic review of population-based cohort studies. Cancer Epidemiol.

[26] Feng F, Tian Y, Liu Z (2016). Clinicopathological features and prognosis of colonic gastrointestinal stromal tumors: evaluation of a pooled case series. Oncotarget.

[27] Reddy RM, Fleshman JW (2006). Colorectal gastrointestinal stromal tumors: a brief review. Clin Colon Rectal Surg.

[28] Fletcher CD, Berman JJ, Corless C (2002). Diagnosis of gastrointestinal stromal tumors: a consensus approach. Hum Pathol.

[29] Stamatakos M, Douzinas E, Stefanaki C, Safioleas P, Polyzou E, Levidou G, Safioleas M (2009). Gastrointestinal stromal tumor. World J Surg Oncol.

[30] Parab TM, DeRogatis MJ, Boaz AM (2019). Gastrointestinal stromal tumors: a comprehensive review. J Gastrointest Oncol.

